# Comparative Evaluation of Fluoride Release and Compressive Strength of Biodentine Modified Using Sodium Fluorosilicate and Hydrofluoric Acid: An In-Vitro Study

**DOI:** 10.7759/cureus.45852

**Published:** 2023-09-24

**Authors:** Velayudham Sekhar, Shekar Shobana, Mahendran Kavitha

**Affiliations:** 1 Conservative Dentistry and Endodontics, Tamilnadu Government Dental College and Hospital, The Tamil Nadu Dr. M.G.R. Medical University, Chennai, IND; 2 Conservative Dentistry and Endodontics, Tagore Dental College, The Tamil Nadu Dr. M.G.R. Medical University, Chennai, IND

**Keywords:** sodium fluorosilicate, fluoride release, hydrofluoric acid, spectrophotometer, biodentine

## Abstract

Introduction

Calcium trisilicate derivatives have widely been used as dentin and enamel substitutes recently. These cements have excellent biological properties, but they do not show fluoride release. The release of fluoride from dental materials is a very important property of a material in restorative applications as fluoride confers antibacterial and anti-cariogenic properties. This study thus attempts to incorporate fluoride in the form of sodium fluorosilicate and hydrofluoric acid into Biodentine to assess its fluoride-releasing properties.

Methods

Biodentine was modified by adding 7% sodium fluorosilicate in the powder and 10% hydrofluoric acid to the liquid. Fluoride release was measured using a sodium 2-(parasulfophenylazo)-1,8-dihydroxy-3,6-naphthalenedisulfonate (SPADNS) spectrophotometer after 1, 3, 7, 14, 21, and 28 days. The compressive strength of the modified material was measured using a universal testing machine.

Results

Fluoride release was found to be higher in the group that had both powder and liquid modified than in groups in which either of the components were modified. The difference was found to be statistically significant (p<0.001).

Conclusion

Incorporation of fluoride in Biodentine is feasible with acceptable release of fluoride without adversely affecting the compressive strength of the Biodentine.

## Introduction

An ideal dentin substitute should have good biocompatibility, long-term impermeability, antibacterial properties, ability to induce hard tissue regeneration, good stability, low solubility, non-absorbency, and ease of handling [1,2]. Biodentine, a tricalcium silicate-based dental material, is free of impurities found in Portland cement derivatives such as MTA. The powder contains tri- and dicalcium silicates, calcium carbonate, and zirconia, and the liquid consists of calcium chloride and water-soluble polymer [3,4]. Biodentine has been used for various vital pulp therapies such as direct and indirect pulp capping in human randomized control trials, clinical trials, and animal and in vitro studies [1,5-10]. Biodentine allows the dentist to achieve biomimetic [11] mineralization in a carious lesion [12]. Biodentine, known as “dentin in a capsule,” has excellent handling properties [13] and better compressive strength [3]. Dentin bridges with the highest average and maximum volume were formed after the use of Biodentin, followed by the use of MTA or Ca(OH)_2_. It can be used as a dentin substitute for posterior restorations [14]. Its good sealing properties, high compressive strength, biocompatibility [15], and short setting time [16-18] support its potential as a restorative material. Although Biodentine is mainly used as a permanent dentin substitute, it can also be used as a temporary enamel substitute for a period of up to six months as caries control restorations [1].

Fluoride is known to have anticariogenic properties [19] and to improve the resistance of enamel and dentin to acid-mediated decalcification. The only property that Biodentine lacks is fluoride release, which is a desirable property of a dental restorative material. Therefore, this study aims to add fluoride to Biodentine in both powder and liquid forms. Sodium fluorosilicate (NaSF) was added to the powder component and hydrofluoric (HF) acid was added to the liquid component. The release of fluoride was studied over a period of up to 28 days by spectrophotometric analysis, and the compressive strength of this modified biomaterial was evaluated using a universal testing machine.

## Materials and methods

This in vitro study was conducted at the Department of Conservative Dentistry and Endodontics, Tamil Nadu Government Dental University and Hospital, Chennai, India. In a pilot study, the setting time and compressive strength of the modified Biodentine in different proportions were investigated. The optimum proportion at which the above properties were not affected was selected for the study at 7 wt% NaSF, which was derived from the pilot study.

Preparation of the modified Biodentine powder containing 7 wt% NaSF (Group A)

The weight of the powder contained in each capsule was accurately measured using a physical balance. The calculation is as follows:

Actual weight of Biodentine = Gross weight of capsule with powder - Weight of empty capsule = 2.25 - 1.53 = 0.7 g. Weight of the 7 wt% NaSF to be added to the Biodentine powder = 0.05 g = 50 mg. The precisely measured amount of 50 mg NaSF is then added to the Biodentine powder capsule and fused at 300 vibrations for three minutes to ensure thorough mixing of the NaSF particles with the Biodentine powder and obtain the modified Biodentine powder.

Preparation of the modified Biodentine liquid containing 10 w/v % 20% HF (Group B)

The 20% HF solution was prepared by diluting 1 mL of commercial 48% HF (Merck, India) with 1.4 mL of deionized water. The liquid component of Biodentine was emptied into an Eppendorf tube. 10 w/v % (20 μL) of 20 % HF was pipetted into the Eppendorf tube containing the liquid Biodentine component using a micropipette to obtain the modified Biodentine liquid.

Preparation by adding modified liquid and powder (Group C)

Preparation of Samples for Fluoride Release Analysis

The powder and liquid components of modified Biodentine were processed in an amalgamator according to the manufacturer's instructions [20]. Cylindrical specimens measuring 5 mm x 5 mm were prepared using polytetrafluoroethylene molds. The sample size was derived from the pilot study as 10 specimens per group each for fluoride release and compressive strength analysis for groups A, B, and C. Ten GIC cylinders and 20 unmodified Biodentine cylinders were prepared as described above and designated as groups D and E, respectively.

The samples were grouped for fluoride analysis as follows:

Group A - Biodentine powder only, modified with 7 wt% NaSF

Group B - Biodentine liquid only modified with 10 wt.% 20% HF

Group C - Biodentine powder modified with 7 wt% NaSF and Biodentine liquid modified with 10 wt% 20% HF

Group D - Glass ionomer cement Fuji IX Extra (positive control)

The specimens were divided into the following groups for compressive strength analysis:

Group A - Biodentine powder only, modified with 7 wt% NaSF

Group B - Biodentine liquid only modified with 10 wt% 20% HF

Group C - Biodentine powder modified with 7 wt% NaSF and Biodentine liquid modified with 10 wt% 20% HF

Group D - Glass ionomer cement Fuji IX Extra

Group E - Unmodified Biodentine (positive control)

Storage of Specimens

After curing the materials, the specimens were removed from the polytetrafluoroethylene molds and stored in 100 mL distilled water in polypropylene containers. Specimens intended for compressive strength analysis were stored at 100% humidity and 37°C.

Assessment of Fluoride Release

The storage media was changed every 24 hours and subjected to fluoride release analysis on the first, third, and seventh days. After the seventh day, the fluoride release was measured cumulatively on the 14th, 21st, and 28th days. The storage medium was used for the analysis of fluoride release using a sodium 2-(parasulfophenylazo)-1,8-dihydroxy-3,6-naphthalenedisulfonate (SPADNS) spectrophotometer. The polypropylene containers were rinsed and replenished with 100 mL of the distilled water between 24 hours to seven days.

Estimation of Released Fluoride

The amount of fluoride released from the samples was quantitatively measured in ppm using the formula:

Fluoride mg/L = mg/L of Fluoride from calibration curve x dilution factor

Conversion of parts per million (ppm) into microgram per square centimeter (μg/cm^2^).

After each reading was taken, the total fluoride released in micrograms was calculated by multiplying the ppm (1 ppm = 1 μg/mL) by the water sample volume (100 mL). The total fluoride was then divided by the area of the sample disc to obtain the fluoride release in micrograms per cm^2^.

Compressive Strength Analysis

Compressive strength analysis was done on groups A, B, C, D, and E. After 24 hours and 21 days, the samples were mounted on cylindrical acrylic resin block using cyanoacrylate and subjected to compressive strength analysis using a Universal testing machine (Instron Corp Canton, MA) at a cross head speed of 0.5 mm/min until the sample gets fractured. The compressive strength values were obtained in MPa.

## Results

The results were tabulated on an Excel sheet and analyzed statistically using SPSS software version 24.0 (IBM Corp., Armonk, NY) (p < 0.05 is considered to be statistically significant).

Statistical analysis

Data were analyzed for normality using the Kolmogrov-Smirnov Test. Data were found to follow a non-normal distribution. Intergroup analysis was done using the Kruskal-Wallis test and pairwise comparison was done using Tukey’s post-hoc test.

Fluoride release

Intergroup and intragroup comparison of F release is shown in Table 1.

**Table 1:** Intergroup and intragroup comparison of F release (μg/cm2) at respective time intervals P-value is significant at the 0.05 level

Time interval	Mean F^—^release (μg/cm^2^)				*P-value
	Group A	Group B	Group C	Group D	
24 HOUR	139.05±6.07	13.33 ± 3.15	97.61 ± 10.90	118.56 ± 4.58	0.0001
3rd day	58.75 ± 4.85	17.52 ± 1.15	80.51 ±7.90	74.42 ± 4.83	0.0001
7th day	49.31 ± 6.04	18.20 ±4.53	97.00 ± 12.64	50.80 ±3.48	0.0001
2nd week	164.21 ±5.72	22.73 ± 2.61	188.89 ±11.53	95.64 ± 5.22	0.0001
3rd week	159.89 ± 8.27	23.67 ± 2.01	258.29 ± 8.73	108.36 ± 4.22	0.0001
4th week	111.88 ± 6.11	14.01 ± 1.85	107.86 ± 5.27	92.85 ± 4.35	0.0001
P-value	0.0001	0.001	0.0001	0.0001	

Post-hoc analysis

24 hours: F¯ release was significantly higher in Group A when compared to Groups B and C (p= 0.001) and significantly higher in Group D when compared to Group C. However, there was no significant difference in the amount of fluoride released when comparing groups A and D, B and C, and C and D.

Third day: F¯ release in Groups C and D was significantly higher when compared to groups A and B (p<0.001).

Seventh day: Group C had significantly higher F¯ release than group B (p<0.000).

14th and 21st days: Groups A and C had significantly higher fluoride release than groups B and D (p=0.001).

**Figure 1:**
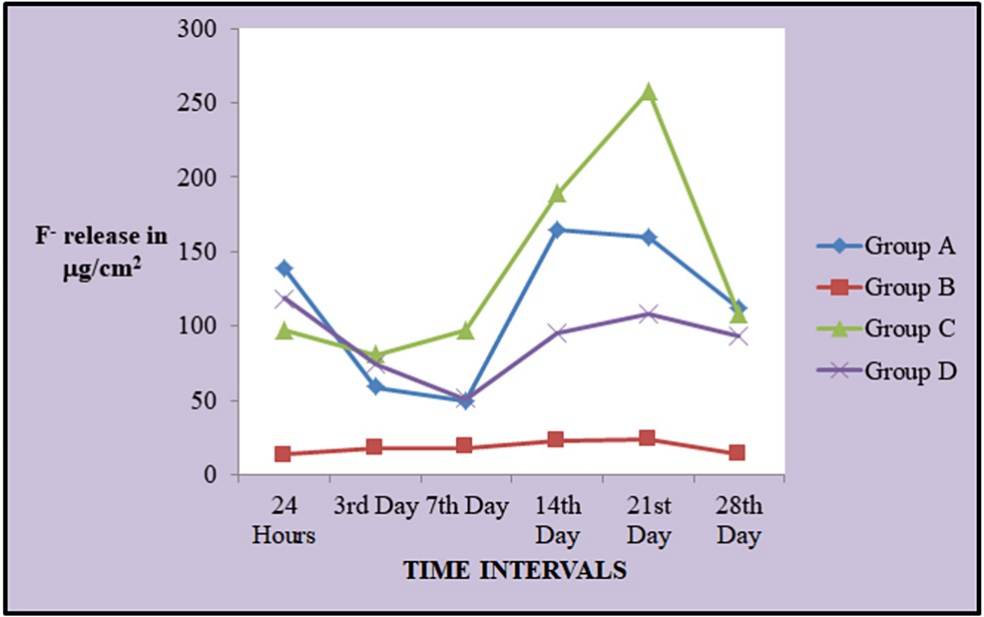
Line diagram showing fluoride release at various time intervals (μg/cm2)

Multiple comparisons between the different groups are shown in Table 2. The trend of fluoride release within the groups at various time points is depicted in Figure 1.

**Table 2:** Tukey post-hoc HSD comparing the fluoride release between the groups at different time intervals * The mean difference is significant at the 0.05 level.

Sample 1-Sample 2	Test statistic	Std. error	Std test statistic	P-value
24 hours				
2-3	-10.200	5.226	-1.952	0.051
2-4	-19.800	5.226	-3.789	<0.001^*^
2-1	30.000	5.226	5.740	<0.001^*^
3-4	-9.600	5.226	-1.837	0.066
3-1	19.800	5.226	3.789	<0.001^*^
4-1	10.200	5.226	1.952	0.051
3 days				
2-1	10.100	5.225	1.933	0.053
2-4	-22.700	5.225	-4.344	<0.001
2-3	-27.200	5.225	-5.205	<0.001
1-4	-12.600	5.225	-2.411	0.016
1-3	-17.100	5.225	-3.272	0.001
4-3	4.500	5.225	.861	0.389
7 days				
2-3	13.400	5.226	2.564	0.010
2-4	-16.600	5.226	-3.176	0.001
2-3	-30.000	5.226	-5.740	<0.001
1-4	-3.200	5.226	-.612	0.540
1-3	-16.600	5.226	-3.176	0.001
4-3	13.400	5.226	2.564	0.010
14 days				
2-4	-10.000	5.226	-1.914	0.056
2-1	20.150	5.226	3.856	<0.001
2-3	-29.850	5.226	-5.712	<0.001>
4-1	10.150	5.226	1.942	0.052
4-3	19.850	5.226	3.799	<0.001>
1-3	-9.700	5.226	-1.856	0.063
21 days				
2-4	-10.000	5.224	-1.914	0.056
2-1	20.000	5.224	3.828	<0.001
2-3	-30.000	5.224	-5.742	<0.001
4-1	10.000	5.224	1.914	0.056
4-3	20.000	5.224	3.828	<0.001
1-3	-10.000	5.224	-1.914	0.056
28 days				
2-4	-14.000	5.225	-2.679	0.007
2-3	-19.500	5.225	-3.732	<0.001
2-1	26.500	5.225	5.072	<0.001
4-3	5.500	5.225	1.053	0.293
4-1	12.500	5.225	2.392	0.017
3-1	7.000	5.225	1.340	0.180

Compressive strength evaluation

The mean 24-hour compressive strength was found to be highest for group E (136.20± 7.22 MPa) followed by group D (97.49±3.26 MPa), group B (85.30 ± 3.93 MPa), group A (72.55 ± 3.34 MPa) and the least compressive strength was for group C (32.70 ± 2.76 MPa). The difference between the groups was found to be statistically significant between all the groups independently (p=0.0001) (Tables 3, 4).

**Table 3:** Compressive strength of various groups at 24 hours (Mpa)

Groups	N	Mean	Std. deviation	Std. error
A	10	72.5590	3.34291	1.05712
B	10	85.3080	3.93139	1.24321
C	10	32.7010	2.76306	0.87376
D	10	97.4970	3.26569	2.31540
E	10	136.7680	7.32195	4.87176

**Table 4:** Post-hoc tests for compressive strength

Groups (I)	Groups (J)	Mean difference (I-J)	Std. error	Sig	
					A	B	-12.74900^*^	1.63190	0.000	
C	39.85800^*^	1.37148							
D	-24.93800^*^	1.47783							
E	-64.20900^*^	2.54531							
B	A	12.74900^*^	1.63190	0.000	
	C	52.60700^*^	1.51955		
	D	12.18900^*^	1.61618		
	E	-51.46000^*^	2.62806		
C	A	-39.85800^*^	1.37148	0.000	
	B	-52.60700^*^	1.51955		
	D	-64.79600^*^	1.35275		
	E	-104.06700^*^	2.47478		
D	A	24.93800^*^	1.47783	0.000	
	B	12.18900^*^	1.61618		
	C	64.79600^*^	1.35275		
	E	-39.27100^*^	2.53526		
E	A	64.20900^*^	2.54531	0.000	
	B	51.46000^*^	2.62806		
	C	104.06700^*^	2.47478		
	D	39.27100^*^	2.53526		

The mean 21 days compressive strength was found to be highest for group E (204.538 MPa) followed by group B (131.51 MPa), group D (113.252 MPa), group A (103.028 MPa) and the least compressive strength was for group C (74.686 MPa). The difference between the groups was found to be statistically significant comparing all the groups independently (p=0.0001) (Tables 5, 6).

**Table 5:** Compressive strength of the groups after 21 days

Groups	N	Mean	P-value
A	5	103.028	0.00001
B	5	131.51	
C	5	74.686	
D	5	113.252	
E	5	204.538	

**Table 6:** Post-hoc test for 24 hours compressive strength of the groups

Groups (I)	Groups (J)	Mean difference (I-J)	Std. error	Sig	
					A	B	-12.74900^*^	1.63190	0.000	
C	39.85800^*^	1.37148							
D	-24.93800^*^	1.47783							
E	-64.20900^*^	2.54531							
B	A	12.74900^*^	1.63190	0.000	
	C	52.60700^*^	1.51955		
	D	12.18900^*^	1.61618		
	E	-51.46000^*^	2.62806		
C	A	-39.85800^*^	1.37148	0.000	
	B	-52.60700^*^	1.51955		
	D	-64.79600^*^	1.35275		
	E	-104.06700^*^	2.47478		
D	A	24.93800^*^	1.47783	0.000	
	B	12.18900^*^	1.61618		
	C	64.79600^*^	1.35275		
	E	-39.27100^*^	2.53526		
E	A	64.20900^*^	2.54531	0.000	
	B	51.46000^*^	2.62806		
	C	104.06700^*^	2.47478		
	D	39.27100^*^	2.53526		

## Discussion

Fluoride is incorporated in the dental material to impart anti-cariogenic properties. There are at least three methods used to combine therapeutic agents such as fluoride into dental materials. These are a simple mixture of water-soluble agents, dispersion of sparingly water-soluble agents, and use of matrix-bound agents [21]. The present study has used NaSF, a sparingly water-soluble agent as a source of fluoride.

NaSF is a complex fluoride salt. Ca++ ions have the ability to hydrolyze NaSF making it suitable for addition with an acidic calcium sulfate solution for remineralization [22]. Owing to the compatibility of NaSF in a calcium-containing milieu, we have chosen this sparingly soluble complex agent as one of the modification agents by incorporating it into the powder component of Biodentine. NaSF has previously been used as a mouth rinse with good cariostatic effects [23].

HF acid is a water-soluble agent that is a good source of fluoride and has been tested as an agent to protect enamel against erosion [24]. Pioch et al. [25] assessed the effect of HF on dentine after exposing dentine to an acid challenge of orthophosphoric acid and found that HF has the ability to retain the smear layer and seal the dentinal tubules. These favorable attributes of HF acid made this agent an ideal candidate for modification of the liquid component of Biodentine.

SPADNS spectrophotometry is a technique employed specifically to detect and quantify the fluoride content of water samples [26]. In this method, a compound of a metal such as aluminum, iron, thorium, zirconium, lanthanum, or cerium reacts with an indicator dye to form a complex of low dissociation constant. This complex reacts with fluoride to give a new complex [27]. In Environmental Protection Agency (EPA) method 340.1, the SPADNS reagent is used, and the color loss is measured at 570 nm (EPA 1998c). Due to the change in the structure of the complex, the absorption spectrum also shifts relative to the spectrum for the fluoride-free reagent solutions. This change can be detected by using a spectrophotometer.

At all estimation time periods, groups A and C showed significantly higher fluoride release when compared to GIC. Fluoride release on the 28th day was comparable with GIC. Dijkman and Arends [28] reported that a monthly cumulative fluoride release of 200-300 μg/cm^2^ is sufficient to completely inhibit enamel demineralization. In the present study, both the powder-only modified as well as component-modified groups showed monthly cumulative fluoride release of around 600 μg/cm^2^ and 700 μg/cm^2^, respectively, which is nearly two to three times the expected levels of fluoride to inhibit demineralization.

The mechanism of fluoride release from modified Biodentine could be similar to the burst effect seen in GIC [21]. This burst effect can happen through surface wash-off, release through pores or cracks, or bulk release. All three study groups showed fluoride release at varying time periods. The powder-only modified Biodentine showed better fluoride release than Glass Ionomer cement which could be attributed to the sparingly water-soluble nature of NaSF, which was used in our study. NaSF has been reported in the literature for modifying the properties of Portland cement [29]. This prompted us to use NaSF as a source of fluoride in Biodentine.

Simila et al. [30] modified Biodentine with bioactive glass and strontium fluoride and achieved higher compressive strength values in the modified Biodentine. Holistically comparing all the modified and unmodified biomaterials used in the present study, none of the materials were found to attain the compressive strength of natural dentin which is around 300 MPa.

In our study, the addition of NaSF and HF acid was found to be much more beneficial in terms of fluoride but compromises the compressive strength. This is in accordance with the study done by Applebaum et al. where the addition of NaSF to mineral trioxide aggregate affected its compressive strength [30]. The compressive strength of all the modified Biodentine groups was lower than the unmodified Biodentine. HF acid-modified Biodentine showed minimal fluoride release probably due to the reaction between CaF2 in the Biodentine liquid with HF. Even though GIC has proved itself to be a biocompatible material with adequate fluoride release, it is acidic and not indicated for deeper cavities with a remaining dentin thickness of less than 1 mm. On the contrary, Biodentine can be used as temporary enamel and permanent dentin replacement, it can be placed directly on exposed pulp. It is also found to be compatible when placed in contact with the gingiva. The only property lacking by Biodentine is fluoride release which made this material a choice for this study.

The mean setting time of group A was 11 min, group B was 15 minutes and group C was nine minutes in contrast to 12 minutes which is the setting time of unmodified Biodentine in the present experimental setting. Thus, powder powder-only modified group decreased, the liquid-only group increased, and both the powder and liquid-modified groups prolonged the setting time of Biodentine. Group C also exhibited mild effervescence during manipulation even though it has not affected the fluoride-releasing properties according to the present study.

The future perspectives of this study are to evaluate the localization of the fluoride ion in the three-dimensional set structure of this modified Biodentine using x-ray diffraction (XRD) and Fourier transform infrared spectroscopy (FTIR), long-term evaluation of compressive strength, flexural strength, the solubility of the material in the oral environment and biocompatibility of thus modified Biodentine. The lack of long-term assessment of the compressive strength and fluoride release, and the lack of assessment of fluoride recharge properties are a few limitations of the study.

## Conclusions

The following conclusions can be drawn from the results of the present study. Fluoridation of Biodentine is achievable by incorporating NaSF in the powder component and hydrofluoric acid in the liquid component. Simultaneous modification of both components of Biodentine showed better fluoride release than the powder-only modification. On the 28th day, the fluoride release of powder only modified Biodentine and both components modified Biodentine were comparable with Glass ionomer cement. The liquid-only modified Biodentine was recorded with only a 32% reduction in compressive strength. The powder-only modified group showed only a 48% reduction in compressive strength Simultaneous modification of both components of Biodentine showed a 77% reduction in 24-h compressive strength whereas the powder-only modified Biodentine showed appreciable fluoride release without much compromise in the compressive strength.

The future scope of the study would be to assess the other biological properties of the modified material like biocompatibility and antimicrobial properties. Within the limitations of this study, it can be concluded that Biodentine powder can be modified to achieve fluoride release without adversely compromising the compressive strength. The powder-only modified Biodentine can be used as a dentine substitute in posterior restorations utilizing the fluoride release properties successfully.
